# *FOXC2* disease-mutations identified in lymphedema-distichiasis patients cause both loss and gain of protein function

**DOI:** 10.18632/oncotarget.9797

**Published:** 2016-06-02

**Authors:** Daniela Tavian, Sara Missaglia, Paolo E. Maltese, Sandro Michelini, Alessandro Fiorentino, Maurizio Ricci, Roberta Serrani, Michael A. Walter, Matteo Bertelli

**Affiliations:** ^1^ Laboratory of Cellular Biochemistry and Molecular Biology, CRIBENS, Catholic University of the Sacred Heart, Milan, Italy; ^2^ MAGI Non-Profit Human Medical Genetics Institute, Rovereto (TN), Italy; ^3^ Department of Vascular Rehabilitation, San Giovanni Battista Hospital, Rome, Italy; ^4^ Medicina Riabilitativa, Azienda Ospedaliero-Universitaria Ospedali Riuniti di Ancona, Torrette, Italy; ^5^ Department of Medical Genetics, University of Alberta, Edmonton, Alberta, Canada; ^6^ Department of Ophthalmology and Visual Sciences, University of Alberta, Edmonton, Alberta, Canada

**Keywords:** primary lymphedema, distichiasis, Foxc2 gene mutations, transcription factor, gain of function, Pathology Section

## Abstract

Dominant mutations in the *FOXC2* gene cause a form of lymphedema primarily of the limbs that usually develops at or after puberty. In 90-95% of patients, lymphedema is accompanied by distichiasis. *FOXC2* is a member of the forkhead/winged-helix family of transcription factors and plays essential roles in different developmental pathways and physiological processes. We previously described six unrelated families with primary lymphedema-distichiasis in which patients showed different *FOXC2* mutations located outside of the forkhead domain. Of those, four were missense mutations, one a frameshift mutation, and the last a stop mutation. To assess their pathogenic potential, we have now examined the subcellular localization and the transactivation activity of the mutated FOXC2 proteins. All six FOXC2 mutant proteins were able to localize into the nucleus; however, the frameshift truncated protein appeared to be sequestered into nuclear aggregates. A reduction in the ability to activate *FOXC1/FOXC2* response elements was detected in 50% of mutations, while the remaining ones caused an increase of protein transactivation activity. Our data reveal that either a complete loss or a significant gain of *FOXC2* function can cause a perturbation of lymphatic vessel formation leading to lymphedema.

## INTRODUCTION

Lymphedema is a dysfunction of the lymphatic system, a disorder characterized by abnormal swelling of one or more extremities due to impaired transport of the lymph [1–3]. This common and debilitating condition affects millions of people worldwide. Lymphedema can be primary (congenital) or secondary (acquired). The prevalence of primary lymphedema has been estimated at 1-5 per 10000 persons (http://www.orpha.net). More than 10 Mendelian forms are known, of which three are considered the major forms: Milroy disease (MD) [MIM 153100], lymphedema-distichiasis syndrome (LDS) [MIM 153400] and Meige disease (MGD) [MIM 153200] [4, 5].

Dominant mutations in the *FOXC2* gene (MIM 602402), coding for the forkhead transcription factor *FOXC2,* cause a form of lymphedema with variable age of onset (range: 7-40 years), often associated with distichiasis [6–8]. *FOXC2* plays a key role in regulation of lymphatic endothelial cells differentiation, in formation of smooth muscle cell layers and in morphogenesis of lymphatic valves. *FOXC2* and *VEGFR-3* act through a common genetic pathway to establish distinct properties of the lymphatic vascular architecture [9].

**Figure 1:**
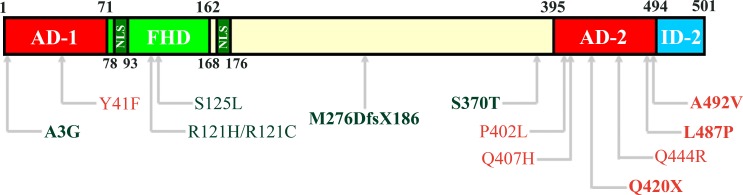
Structural domains of FOXC2 protein In the schematic representation of FOXC2 (amino acids 1-501), the Activation Domain 1 (AD-1) is located between amino acid 1 and 71. The Forkhead Domain (FHD, amino acids 71-162) is the DNA-binding region and contains also the first Nuclear Signal (NLS, amino acid 78-93). The second NLS is located between amino acid 168 and 176. In the C-terminal region, there are the Activation Domain 2 (AD-2, amino acids 395-494) and the Inhibitory Domain 2 (ID-2, amino acids 494-501). Furthermore, the localization of mutations analyzed by functional studies are reported in the scheme [Red: activating mutations; Green: inactivating mutations; Bold, mutations described by Michelini et al. (2012); Regular, mutations described by Berry et al. (2005) and van Steensel et al (2009)].

*FOXC2* maps to 16q34.3 and it produces a 2.2 kb transcript with a 1.5 kb single exon coding region The FOXC2 protein contains 501 amino acids (Figure 1). The most characterized region in the gene is the forkhead DNA binding domain (FHD, amino acids 71 to 162), containing also the nuclear signal 1 (NLS1, amino acids 78-93). At the N-terminal there is the transactivation domain 1 (AD-1) starting from the first amino acid until the FHD (amino acid 71). In the central region of FOXC2 protein, after the NLS2 (amino acids 168-176), some phosphorylation and SUMOylation conserved sites have been recently identified, conferring to this sequence a negative regulative role [10, 11]. Finally, in the C-terminal sequence, a second transactivation domain has been described (AD-2, amino acids 395-494) and an inhibitory region (ID-2, amino acids 495-501) [10, 12].

In LD patients, almost 70 different *FOXC2* mutations have been reported to date, scattered all along the coding sequence (http://www.hgmd.cf.ac.uk). The majority of *FOXC2* mutations are small insertions or deletions and nonsense mutations causing truncated proteins, which have been hypothesized to be responsible for a haplo-insufficiency condition [7,8]. The *FOXC2* haplo-insufficient state is associated to hyperplasia and distichiasis in mice [13]. *FOXC2*−/− mice have been found to exhibit abnormal pericyte recruitment to the lymphatic capillaries and valve agenesis in the collecting vessels, indicating that FOXC2 is essential for the formation of a pericyte-free lymphatic network and lymphatic valve development [14]. Moreover, it was noted that the craniofacial, cardiovascular, and skeletal abnormalities sometimes associated with lymphedema-distichiasis syndrome in humans, had previously been shown to be fully penetrant in homozygous *FOXC2*-null mice [15, 16].

The pathogenesis of LD was assumed to be associated with *FOXC2* haplo-insufficiency also in humans, until some *FOXC2* missense mutations, identified in LD patients, were found to cause a gain of protein function [17]. Approximately 24 % of *FOXC2* variations are missense mutations of which very few have been functionally investigated. Two mutations located inside the forkhead domain impaired the DNA-binding and transcriptional activation ability of FOXC2 protein [18]; in contrast, four mutations located outside the forkhead domain caused a gain of function [17]. Moreover, no data are available on the transactivation properties-function of FOXC2 truncated proteins due to stop mutations or small insertions/deletions preserving at least half of the protein sequence, although together they represent almost 50% of *FOXC2* variations. We therefore performed functional characterization of *FOXC2* mutations located in different protein domains to provide novel information on structure-function relationships of the FOXC2 transcription factor and to get further insight into the disease-causing mechanism of lymphedema.

## RESULTS

### Patient descriptions

Genetic evaluation of LD patients has been previously reported [19]. Molecular and clinical findings are shown in Table 1. All but two of the patients (P1 and P3) presented with distichiasis. No evidence of heart defects, cleft palate, extradural cysts or other distinctive characters have been reported, with the exception of P4. Finally, no superficial and deep venous insufficiency and recurrent erysipelas have been observed. A detailed clinical description of LD patients is reported in Supplementary material 1.

**Table 1:** Summary of patients' molecular and clinical date

Patient	Mutation	Protein domain	Transcriptional. activity of mutated protein*	Age of onset	Lymphedema	Lymphatic hypoplasia or hyperplasia**	Distichiasis***	Cardiac defects/other clinical findings
1 M (69 y)	A3G	AD-1	10%	50 y	bilateral, greater on right	hyperplasia	no	no
2 M (46 y)	M276fs	Central region	16%	12 y	bilateral, greater on right	hyperplasia	yes	no
3 F (19 y)	S370T	Central region	73%	14 y	bilateral	uncertain evaluation	no	no
4 M (28 y)	G420X	AD-2	257%	19 y	bilateral, greater on right	hypoplasia	yes	Bicuspid aortic valve/ extradural cyst
5 F (34 y)	L487P	AD-2	172%	26 y	bilateral, greater on left	hypoplasia	yes	no
6 M (30 y)	A492V	AD-2	182%	26 y	left unilateral	hypoplasia	yes	no

* The percentage of transcriptional activity of FOXC2 mutated proteins was calculated considering FOXC2 wild type signal as 100% of activity (Luciferase Reporter assay)

** Lymphatic hypoplasia or hyperplasia by lymphoscintigraphy evaluation performed by two clinicians independently, referring on lymph nodes up take of radiocolloid and on the presence or absence of dermal backflow

*** Patients were examined by an ophthalmologist using a slit lamp for evidence of distichiasis

M: Male; F: Female

### Functional characterization of FOXC2 mutations

The mutations analysed in this study were all localized outside the forkhead domain, in different regions of FOXC2 protein (Figure 1). The evaluation of missense mutations by bio-informatic prediction tools and the homology comparison between different species are reported in Supplementary Figure 1. To verify whether these mutations can affect FOXC2 function by reducing mRNA or protein stability, altering nuclear localization, or impairing transactivation activity, we subcloned wild-type *FOXC2* cDNA into the pcDNA3.1/NT-GFP-TOPO TA expression vector and then performed site-direct mutagenesis to generate the *FOXC2*-GFP plasmids with the disease causing mutations.

**Figure 2:**
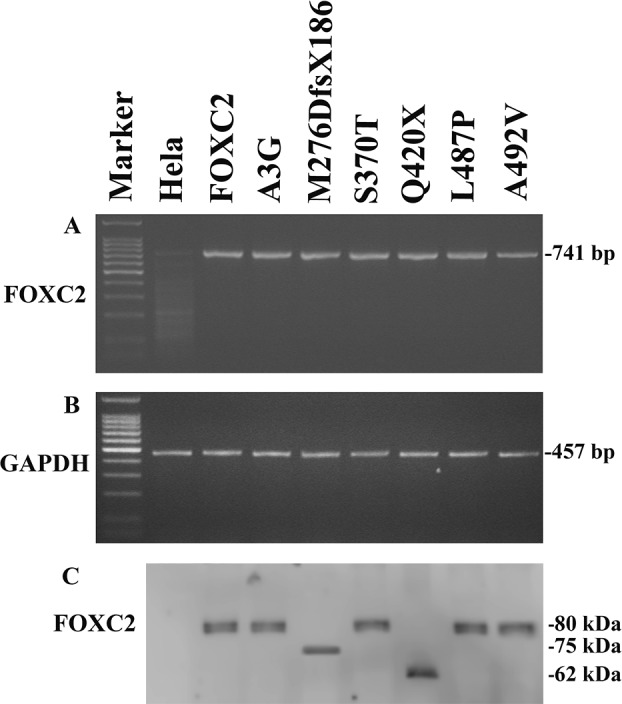
Exogenous FOXC2 expression of mutant recombinant plasmids **A.** RT-PCR detection of exogenous FOXC2 (741-bp product) and **B.** GAPDH (457-bp product) in HeLa transfected cells with p*FOXC2*-GFP, p*FOXC2*(A3G)-GFP, p*FOXC2*(M276DfsX186)-GFP, p*FOXC2*(S370T)-GFP, p*FOXC2*(Q420X)-GFP, p*FOXC2*(L487P)-GFP and p*FOXC2*(A492V)-GFP (lanes 3-9); lane 1 refers to DNA molecular weight marker and lane 2 refers to non-transfected HeLa cells. **C.** Western blotting analysis of exogenous FOXC2 protein expression in HeLa cells transfected with plasmids reported in (A) and in (B).

When transfected into HeLa cells, wild type and mutant plasmids showed similar and stable *FOXC2* mRNA levels (Figure 2A). Exogenous *FOXC2* expression was detected by RT-PCR analysis with primers that selectively amplify the cDNA obtained from the recombinant plasmids. Endogenous *GAPDH* RT-PCR products were detectable in similar amounts in all samples tested and were used to normalize FOXC2 RT-PCR values for each sample (Figure 2B).

In agreement with RT-PCR results, western blotting analysis showed stable expression of all recombinant proteins (Figure 2C), that appeared to be phosphorylated with the only exception being p.M276DfsX186. There were no apparent differences in phosphorylation between wild type and FOXC2 proteins carrying missense mutations as detected in electrophoretic mobility assays. The Q420X FOXC2 protein was also phosphorylated, however it presented a different migration profile in comparison with wild type protein due to the mutation resulting in a truncated FOXC2 protein. Finally, the p.M276DfsX186 FOXC2 protein was detected as a single band on western blots, and thus appears not to be phosphorylated. The p.M276DfsX186 FOXC2 protein is larger than the p.Q420X mutant protein, since the former consists of 462 amino acids (276 of FOXC2 protein plus 186 due to the frameshift) while the latter of 420 amino acids.

**Figure 3:**
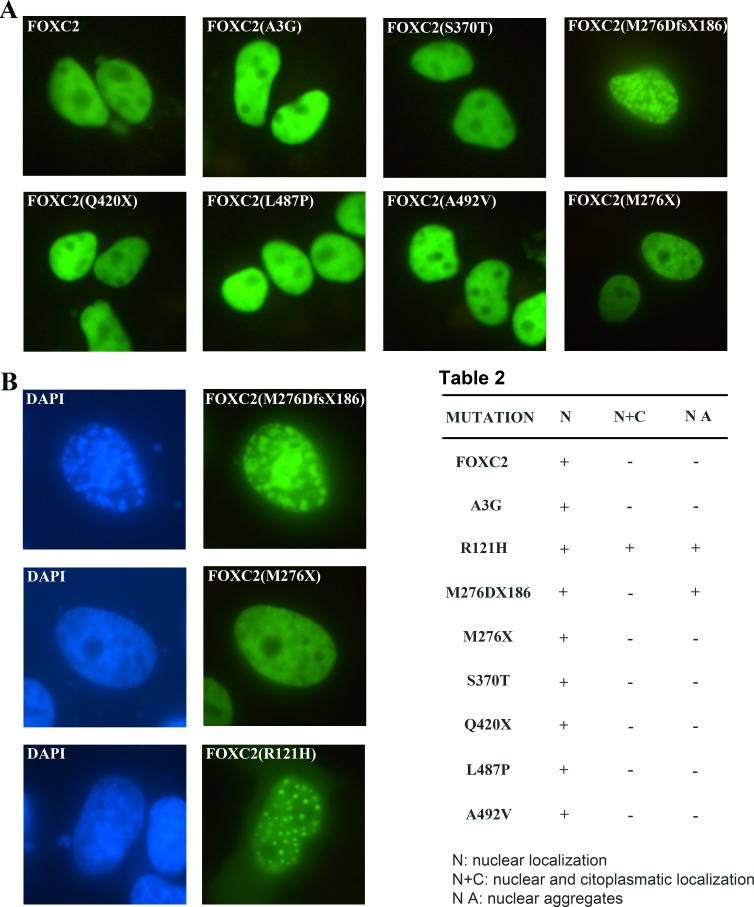
Transient transfection of FOXC2 mutant proteins in HeLa cells HeLa cells were transiently transfected with one of the following plasmids: p*FOXC2*-GFP, p*FOXC2*(A3G)-GFP, p*FOXC2*(S370T)-GFP, p*FOXC2*(M276DfsX186)-GFP, p*FOXC2*(Q420X)-GFP, p*FOXC2*(L487P)-GFP, p*FOXC2*(A492V)-GFP and p*FOXC2*(M276X)-GFP. After 24 h, cells were fixed and stained with DAPI. Fluorescence of FOXC2-GFP fusion proteins is in green. **A.** The nuclear localization of all FOXC2 mutant proteins was detected by direct immunofluorescence analysis of plasmids tagged with GFP; images were at 40X magnification. **B.** Immunofluorescence evaluation of HeLa cells transiently transfected with the following recombinant plasmids: p*FOXC2*(M276DfsX186), p*FOXC2*(M276X) and p*FOXC2*(R121H). The M276DfsX186 mutation causes FOXC2 protein aggregates, as well the R121H mutation. In green, FOXC2 proteins tagged with GFP and in blue, cellular nuclei stained with DAPI. More than 200 cells were counted to detect nuclear versus cytoplasmatic localization. Magnification:100X.

To assess whether mutations affected the ability of FOXC2 proteins to localize properly into the nucleus, HeLa cells were transfected with wild type and mutant plasmids. We included also the *FOXC2*(R121H) mutation-containing plasmid as a control in order to allow an accurate comparison of intracellular distribution, since the FOXC2(R121H) protein has been previously demonstrated to display a defect in nuclear localization, with partial cytoplasmic signal [18]. As expected, immunofluorescence analysis revealed that wild type FOXC2 was localized exclusively into the nucleus and displayed a homogeneous distribution (Figure 3A). All disease mutations did not affect FOXC2 nuclear localization; moreover, no cytoplasmic signal could be detected for any of them (Figure 3A; Table 2). However, the transfection of *FOXC2*(M276DfsX186) recombinant plasmid caused the production of nuclear aggregates (Figure 3B). A similar effect was observed utilizing COS7 cells (Supplementary Figure 2). Also the *FOXC2*(R121H) plasmid, in our system, induced intranuclear protein aggregation (Figure 3B). In contrast, the FOXC2(M276X) truncated protein revealed a homogeneous fluorescence signal, very similar to that of wild type (Figure 3B).

**Figure 4:**
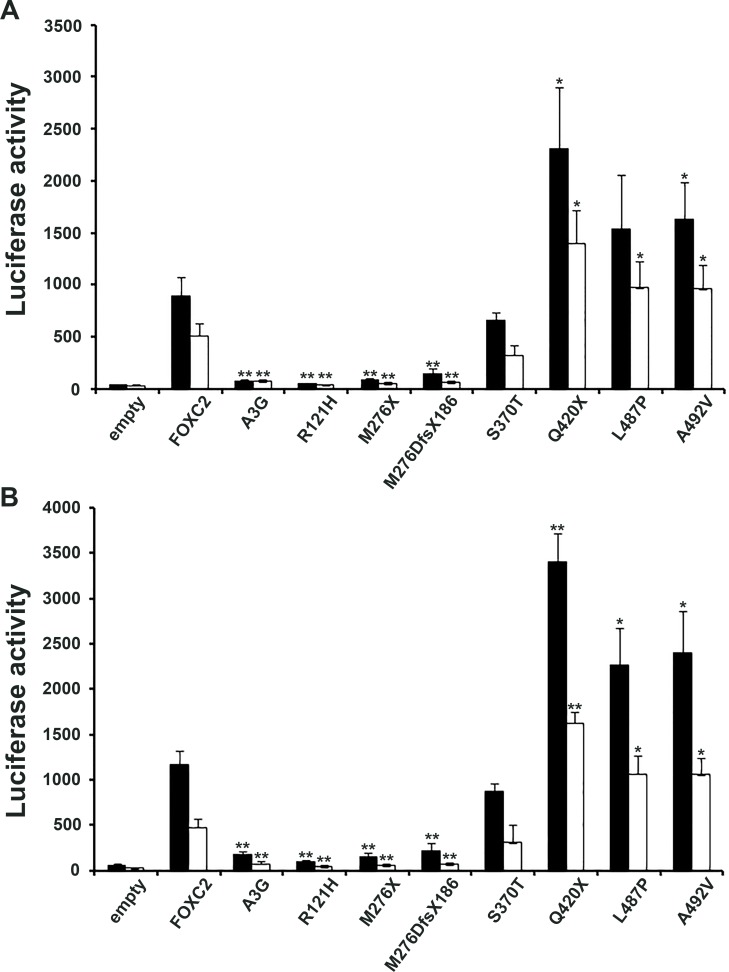
Transactivation activity of wt and mutant FOXC2 proteins Luciferase assays were used to measure protein transactivation abilities. **A.** Transactivation activity of wt and mutant FOXC2-GFP fusion proteins compared with that of empty vector in HeLa (black squares) and COS7 (white squares) cells indicated as fluorescence units (vertical axis). **B.** Transactivation activity of wt and mutant FOXC2 proteins (proteins without any tag) compared with that of empty vector in HeLa or COS7 cells. Thick bar: mean value, error bar: SD. Significant differences were detected by using Student's t-test. P-values of <0.05 and of <0.01 were considered to be significant and indicated with “*”and “**”, respectively.

FOXC2 is known to act as a transcription factor; therefore, the ability of FOXC2 mutant proteins to stimulate the expression of a reporter gene was tested in HeLa and COS7 cells. GFP-FOXC2 fusion proteins recapitulated the transcriptional activity of FOXC2 mutant proteins expressed without an exogenous tag, in both HeLa and COS7 cells (Figure 4A and 4B). The Q420X and A492V mutations displayed significantly increased FOXC2 transcriptional activity in comparison with wild type protein in all the experimental conditions tested (Figure 4). The L487P mutation showed significance significant gain of protein function in 3 out 4 of luciferase assays. In contrast, the A3G and M276DfsX186 mutations were not able to activate the luciferase reporter vector above background levels (Figure 4). These mutations appeared to be similar to R121H variation, as they rendered the FOXC2 transcription factor totally inactive. Finally, the S370T mutation also decreased transcriptional activity but only to almost 30% of that of FOXC2 wild type protein (Figure 4). Again, mutation R121H served as methodological negative control in our experiment, since it had previously found to be transcriptionally inactive in luciferase assays [17].

## DISCUSSION

Despite the great importance of the FOXC2 transcription factor in human pathophysiology and the increasing number of different mutations identified in patients with lymphedema, the molecular consequences caused by *FOXC2* gene variations are almost entirely unknown [20–22]. Only a small percentage of FOXC2 mutations have been functionally investigated so far (7/67, ~ 10%); all of which are missense mutations [17, 18]. In this report, we have analyzed the functional consequences of six FOXC2 disease mutations identified in LD patients. The A3G mutation is the FOXC2 mutation closest to the ATG starting codon identified to date. The p.A3G variation was considered “probably damaging” by Polyphen-2 and “deleterious” by Sift. This mutation is localized in the highly conserved AD-1 region of the FOXC2 protein. Although the A3G mutation does not change the amino acid charge, both bioinformatics prediction and our functional data reveal that it is a pathogenic mutation, drastically reducing FOXC2's capability to activate expression of target genes. It is difficult to explain the biological dysfunction caused by single site amino acid replacement that does not result in charge-changing, unless it is assumed that this mutation decreases the ability to adopt the FOXC2 wild type conformation. As the complete 3D structure of FOXC2 protein is currently not available, it is not currently possible to exam the 3D structure of the A3G mutant protein. Interestingly, a significant increase of function was previously reported in FOXC2 protein carrying the p.Y41F mutation, also located within the AD-1 domain [17]. Taken together, these results indicate that missense mutations within the FOXC2 AD-1 can result in either the loss or gain of function.

We examined three FOXC2 mutations located within the AD-2 region: two missense (p.L487P and p.A492V) and one nonsense (p.Q420X) mutations. All three AD-2 mutations significantly increased FOXC2 transcriptional ability. Surprisingly, the p.Q420X displayed the highest level of activation, despite the truncated protein lacked more than half of AD-2 sequence and the entire ID-2 region. Consistent with these findings, Van Steensel et al. also reported a gain of function for FOXC2 missense variations localized inside AD-2 domain. We did not detect any evidence of mRNA or protein stability alterations resulting from FOXC2 AD-2 mutations. Thus, it is possible that AD-2 mutations exert their influence by modifying the tridimensional structure of the C-terminal domain of the FOXC2 protein and/or changing the ability of FOXC2 to interact with regulatory proteins. In the central region of *FOXC2,* we have identified the p.M276DfsX186 mutation (in Patient 2) which leads to the formation of a truncated protein lacking of 235 amino acids in the C-terminal region, and with the addition of 186 nonsense amino acids due to the frameshift (Figure 1). This mutated protein did not have an impaired ability to localize into the cell nucleus, however, the FOXC2 p.M276DfsX186 mutant protein condensed into protein aggregates. In contrast, the p.M276X FOXC2 mutation localized to the nucleus without aggregating. We hypothesize that the presence of the extra 186 nonsense amino acids due the frameshift mutation resulted in the protein aggregation. Never the less, both p.M276DfsX186 and p.M276X FOXC2 mutations caused the complete loss of protein function, as might be expected for mutations that result in mutant proteins lacking about one half of the FOXC2 protein.

Finally, the S370T missense mutation, located in the terminal sequence of central region (Figure 1), resulted in a slight decrease of the ability of FOXC2 to trans-activate genes. Interestingly, S367 has been recently identified as a phosphorylation site for FOXC2 [11]. It thus cannot be excluded that S370T mutation partially influences the phosphorylation process of S367, triggering changes in the FOXC2 transcriptional program.

To date, it is not possible to extrapolate general conclusions about the relationship between FOXC2 activity and the protein variation position, because of the very limited number of different types of mutations (missense, nonsense and frameshift) that have been functionally tested. However, data obtained so far indicate that missense mutations in FHD and AD-2 domain might represent an exception, causing always a loss and a gain of FOXC2 function, respectively. More data are needed to highlight possible correlation between mutation position and function in other FOXC2 protein domains.

Nowadays, it is possible to estimate the theoretical pathogenic effects of missense mutations utilizing bioinformatics tools. However, the usefulness of these programs in predicting the real contribution of a mutation becomes limited when they provide conflicting data (see Supplementary Figure 1B, for p.A492V mutation) [23]. Moreover, current bioinformatics tools are not able to predict whether a mutation causes a gain or a loss of function. Only functional studies indicate how mutations impair protein ability, providing data that could be clinically relevant. This is true for genes encoding metabolic enzymes critically important for human pathologies [24–27] and, even more, for transcription factors, like *FOXC2*, that are responsible for regulating the expression of a broad range of genes both during development and in adult tissues [12, 17, 28].

From our data it appears that no easy relationships can be established between the level of transcriptional activity of FOXC2 mutated proteins and the degree of pathology. Never the less, some clinically relevant information appears to be revealed by our analyses. After comparing the lymphoscintigraphy of the two patients harboring a considerable loss of function mutations (P1 and P2) with the others carrying a gain of function mutations (P4, P5 and P6), we noted some differences in the uptake of the radiopharmacon by inguinal lymph glands and its reflux back down into the lower leg. In particular, P1 and P2 patients showed a lymphoscintigram profile/images compatible with a hyperplasia of lymphatic vessels (with dermal backflow), while P4, P5 and P6 patients reports resembled more a hypoplastic condition (Supplementary Fig 3). While, lymphangiography is the best method to distinguish between hyper and hypoplasia [8, 17], our investigation indicate that lymphoscintigraphy may provide additional indication in those cases in which a considerable difference of gain or loss of function can be detected. In patient P3, where, a mild hyperplasia might be predicted since the S370T mutation causes a slight loss of transcriptional activity, it was not possible to highlight the modest increase of new lymphatic vessel formation using lymphoscintigraphy.

Based upon clinical and molecular data, therefore, we can hypothesize that an increased activity of FOXC2 results in disregulated expression of proteins essential for normal development of lymphatic system, causing hypoplasia. In contrast, decreased FOXC2 transcriptional activity appears to be associated with a hyperplastic condition. Our hypothesis on hypo or hyperplasia and activating versus inactivating mutations is consistent with previously reported results [17, 18] and with the hyperplastic condition of haplo-insufficienct FOXC2+/− mice who present with abnormal lymphatic drainage, increased number of lymph nodes and lymph backflow [13, 14]. Should transgenic mice carrying FOXC2 activating mutations become available, it would be interesting to discover if they develop a hypoplastic lymphatic system as predicted by our model.

The association of FOXC2 mutation function and distichiasis is less clear however. Our patients with activating mutations presented with distichiasis, in contrast, 2 out of 3 patients with inactivating mutation did not. From these clinical findings, it might be tempting to speculate that distichiasis is associated with hypoplasia rather than hyperplasia. However, additional larger clinical studies are required to definitely elucidate correlations between *FOXC2* different mutations and the morphologic changes of lymphatic system, and with distichiasis.

In conclusion, we have analyzed the molecular consequence of *FOXC2* mutations identified in six Italian families with lymphedema-distichiasis, providing evidences of patients with inactivating versus activating mutations of the FOXC2 transcription factor. During development, transcriptional programs of lymphatic endothelial cells are established by networks of transcriptional factors, among which FOXC2 is one of the most important. FOXC2 acts downstream of VEGFR-3 signaling pathway and is essential for the formation of pericyte-free lymphatic network. Moreover FOXC2 regulates the expression of genes essential for all steps of lymphatic valve morphogenesis and maintenance, such as connexins CX43 and CX47 [4, 5, 29, 30]. Our data indicate that either loss or gain of FOXC2 function is deleterious, causing hyperplasia or hypoplasia respectively in patients with primary lymphedema-distichiasis. We predict that any change in the activity level of FOXC2 causes an unbalanced expression of molecular regulators of lymphangiogenesis, a highly orchestrated process not yet fully understood.

Finally, recent studies show that FOXC2 overexpression is involved in cancer progression, inducing epithelial mensenchymal transition (EMT) [31, 32]. LDS patients carrying FOXC2 gain of function mutations should be carefully monitored in order to verify whether they present an higher cancer susceptibility and to ensure the earliest identification of possible lesions.

## MATERIALS AND METHODS

### Cloning the *FOXC2* cDNA and generation of site-directed mutagenesis plasmids

*FOXC2* cDNA was amplified by PCR using DNA clone ID 32938 (Thermo Scientific) as template and the following primers: FOXC2-F 5′-ATGCAGGCGCGCTACTCC-3′ and FOXC2-R 5′-TCAGTATTTCGTGCAGTCGTAGGAG-3′. The PCR product was subcloned into pcDNA3.1/NT-GFP-TOPO from Invitrogen to produce NT-GFP-FOXC2, expressing FOXC2 with GFP at the N-terminus. Point mutations were performed using the Phusion Site-Directed Mutagenesis Kit (Thermo Scientific). Mutations in *FOXC2* cDNA were introduced using the following primers: A3G forward 5′-ATGCAGGGGCGCTACTCCGTGT-3′ and reverse 5′-ACACGGAGTAGCGCCCCTGCAT-3′; R121H forward 5′- GCAGAACAGCATCCACCACAACCTCTCGC -3′ and reverse 5′- GCGAGAGGTTGTGGTGGATGCTGTTCTGC -3′; S370T forward 5′-GTCGCCCCTGACCGCTCTCAACC-3′ and reverse 5′-GGTTGAGAGCGGTCAGGGGCGAC -3′; Q420X forward 5′- GCCGCGGCGTAGGCGGCCT-3′ and reverse 5′- AGGCCGCCTACGCCGCGGC -3′; L487P forward 5′-ACGCCGCCTCCCTATCGCCAC-3′ and reverse 5′-GTGGCGATAGGGAGGCGGCGT-3′; A492V forward 5′-TCGCCACGCAGTCCCCTACTCCT-3′ and reverse 5′-AGGAGTAGGGGACTGCGTGGCGA-3′.

In order to obtain the c.826-827delAT FOXC2 mutation (M276DfsX186) were used the following two couple of primers and two rounds of mutagenesis (see supplementary materials): forward(1) 5′- CAGCGTGGAGAACATCTGACCCTGCGAACGTC-3′ and reverse(1) 5′- GACGTTCGCAGGGTCAGATGTTCTCCACGCTG-3′ and forward(2) 5′- CAGCGTGGAGAACATCGACCCTGCGAACGTC-3′ and reverse(2) 5′- GACGTTCGCAGGGTCGATGTTCTCCACGCTG-3′.

NT-GFP-*FOXC2* (wild type) and NT-GFP-*FOXC2* mutant plasmids were used as template to amplify *FOXC2* control and mutants cDNA sequences with FOXC2-F and FOXC2-R primers (reported above). The PCR products were subcloned into pcDNA3.3-TOPO (Life Technologies) to produce control or mutant FOXC2 proteins without any tag. All final expression constructs were sequenced to verify that no additional mutations were introduced.

### RT-PCR analysis of FOXC2 recombinant mRNAs in HeLa cells

HeLa cells were transiently transfected with NT-GFP-FOXC2 recombinant plasmids using the TurboFect transfection reagent, according to the manufacturer's protocol (Thermo Scientific). After 48 h, cells were carefully washed with PBS, total RNA was isolated with TRIzol (Invitrogen) and 1 μg was converted to cDNA by RT-PCR using random hexamers (0.5 mg), 400 units of MMLV-RT, 1.6 mM total dNTPs, 20 units of Rnasin and 0.4 mM dithiothreitol, in a 50 μl reaction solution containing 10× RT Buffer. Before reverse transcription, RNA was treated with DNase in order to eliminate DNA contamination [33]. RT-PCR reactions were optimized for *FOXC2* and *GAPDH* gene, in order to avoid saturation: regression curves assaying different amounts of cDNAs (corresponding to different mRNA concentrations) and different number of cycles of amplification were performed (Supplementary Figure 4). Thirty nanograms of cDNA were used to perform PCR amplification using GFP-F (5′-CGACACAATCTGCCCTTTCG-3′) and FOXC2-intR (5′- CCGGGGGCGGCTCCTTG -3′) primers designed to produce a 741 bp fragment of the GFP-FOXC2 transcript. PCR conditions were as follows: denaturation at 94°C for 10 min, annealing at 59°C for 30 sec and extension at 72°C for 30 sec for the first round, denaturation at 94°C for 30 sec, annealing at 59°C for 30 sec and extension at 72°C for 30 sec for 26 cycles; denaturation at 94°C for 30 sec, annealing at 59°C for 30 sec and terminal extension at 72°C for 10 min for the last cycle. The PCR products were electrophoresed on a 2% agarose gel containing ethidium bromide. The concentration value of FOXC2 gene was normalized versus the constant level of GAPDH in each sample. Images of gels were acquired (Bio-Rad Gel Doc 2000, Bio-Rad, Hercules, CA, USA) and scanned using Quantity One Analysis software (Bio-Rad). This software allows the detection of the mean value of each band (i.e. the mean intensity of the pixels inside the volume of band) and the concentration of PCR products, calculated from the standards included in each gel [34].

### Localization assay of FOXC2 mutant proteins in HeLa and COS cells

For transient transfections, HeLa and COS cells were cultured on glass coverslips in Dulbecco's modified Eagle's medium supplemented with 10% fetal bovine serum (FBS) and allowed to adhere overnight. The next day, the cells were transiently transfected with recombinant NT-GFP-FOXC2 plasmids using the TurboFect transfection reagent. After 24 h, cells were fixed stained with DAPI, and examined with a Leica MB5000B microscope equipped with 40X and 100X Fluorart oil immersion objectives.

### Western blot analysis of FOXC2 proteins expression in transfected HeLa cells

HeLa cells were transiently transfected with recombinant NT-GFP-FOXC2 plasmids as described above. Four flasks for each type of recombinant plasmids was transfected. After 48 h, all flasks were evaluated for transfection efficiency. Only samples reaching 80% of transfection efficiency were used to prepare protein cell extracts. Cell extracts were prepared from confluent cultures grown in serum-containing medium. After extensive washing with Dulbecco's phosphate-buffered saline (DPBS), cells were recovered by scraping with a rubber policeman in 300 μl of 0.05% (wt/vol) SDS. The total protein concentration of cell extracts was quantified using a Coomassie (Bradford) Protein Assay Kit (Pierce). Proteins (10 μg/well) were separated by electrophoresis on 8% SDS-polyacrylamide gel (Bio-Rad), transferred to a nitrocellulose membrane (Pierce) and then immunoblotted using a rabbit monoclonal antibody (dil. 1:5000) raised against green fluorescent protein (Invitrogen) and a mouse monoclonal antibody (dil: 1:5000) to GAPDH (Abnova). Specifically bound immunoglobulins were detected using the SuperSignal West Pico Complete Detection Kit (Pierce) containing ImmunoPure Peroxidase Conjugated Goat anti-Mouse and anti-Rabbit IgG (dil 1:20.000). Experiments were repeated three times.

### Luciferase assays

HeLa and COS cells were co-transfected with 20 ng FOXC1 luciferase reporter [35] and 100 ng wild type or GFP-FOXC2 recombinant plasmids using TurboFect reagent (Thermo Scientific), according to the manufacturer's recommendations in a 96 well format. Each transfection was performed in triplicate. To measure FOXC2 activity, all transfected cell lines were incubated for 40 h, prior to lysis of the culture and addition of substrate from the Britelight plus kit (PerkinElemer). For each clone, the average expression level in Fluorescent Units (FU) (from Photinus pyralis, reporter) was calculated after correction for transfection efficiency. This was obtained as measure of GFP fluorescence in the cells transfected with NT-GFP-FOXC2 recombinant plasmids [36].

The same experimental conditions were used for transient transfection of wild type or FOXC2 mutant plasmids subcloned into pcDNA3.3, a mammalian expression without tag. The dual-luciferase assays were used to obtain sequential quantification of both Photinus pyralis luciferase (reporter vector) and Renilla reniformis luciferase (control vector), as previously described (35). Luminescence detection for all transfected plasmids was performed using the Glomax luminometer (Promega). Reactions were replicated three times, using the Promega Dual Luciferase Assay kit (Promega).

### Statistical and bio-informatic analysis

The statistical analysis of quantitative data of RT-PCR and of Luciferase assays was made using SPSS v.19 package (SPSS, Chicago, IL, USA). The values were compared using Student's t-test. A P-value of ≤0.05 was considered to be statistically significant.

The effect of amino acid substitutions on protein function was predicted using ClustalW, SIFT and PolyPhen software. A multiple sequence alignment of mammalian FOXC2 proteins was used as imput for ClustalW. The NCBI reference sequence (FOXC2 protein NCBI accession number: NP_005242.1.) of the human FOXC2 protein was used as the input for SIFT and PolyPhen, with default query options.

## SUPPLEMENTARY MATERIALS


