# Supplementary material for: FOXC2 disease-mutations identified in lymphedema-distichiasis patients cause both loss and gain of protein function
Source: Oncotarget. 2016 Jun 2;7(34):54228–39. doi: 10.18632/oncotarget.9797 (PMC5342337; doi:10.18632/oncotarget.9797)
Supplement: Supplementary file 1 [file oncotarget-07-54228-s001.pdf]

FOXC2 disease-mutations identified in lymphedema-distichiasis patients cause both loss and gain of protein function

Supplementary Material

Clinical description of LD patients

Patient 1- A3G mutation

Patient 1, a 69-year-old man, developed bilateral lymphedema, greater on right, at the age of 50. Lymphoscintigraphy, performed in 2009, showed a moderate slowing of radiocolloid return flow along lymphatic vessels of left lower limb, with good visualisation of inguinal lymph nodes. In the late phase of the test, weak visualisation of homolateral iliac lymph nodes was possible. Visualisation of lymphatic ducts and lymph nodes of the right lower limb was absent, even in the late phase; there was evident dermal backflow.

Patient 2- M276DfsX186 mutation

This male patient, age 46 years, developed bilateral lymphedema at the age of 12. Lymphoscintigraphy, performed in 2003, showed very slow movement of the tracer in both limbs in the early phase, with partial visualisation on the left, associated with dermal backflow, and complete absence of visualisation on the right, even in the late phase of the investigation. When the test was repeated after physical therapy, rapid passage of the radiocolloid along the lymphatic ducts of the lower limbs was observed, showing that the lymphatic circulation was present and associated with dermal backflow in both limbs. Moreover, the lymph nodes were found positive after therapy, whereas previously they were silent. These findings might be consistent with a hyperplastic condition, characterized by an increased number of lymphatic vessels, sometimes of large dimension, causing lymph reflux, known as dermal backflow (see also patient 1).

Patient 3- S370T mutation

Female patient 3, age 19 years, developed bilateral lymphedema at the age of 14. Lymphoscintigraphy showed absence of lymphatic drainage bilaterally of the superficial circulation in all phases of the investigation.

Patient 4- Q420X mutation

Male patient 4, 28 years of age, developed lymphedema in right lower limb at age 19 years, followed by left lower limb with progression up to the knee. At age 22 years, he complained of inguinal pain after physical exercise; a bilateral inguinal ultrasonography showed some subcutaneous lymph nodes of increased volume. The last echographic examination (2014) showed two large right inguinal lymph nodes; lymphedema remained more severe in the right lower limb, in agreement with lymphoscintigraphic findings. Indeed, lymphoscintigraphy showed bilateral slowing of lymph drainage, more evident on the right, where collateral circulation was evident; dermal backflow and anomalous lymph node stops were absent. Scintigraphic imaging definition of the left inguinal and lumbar lymph nodes was normal, whereas that of the right inguinal, iliac and lumbar lymph nodes was reduced, even several hours after injection. This finding might suggest a hypoplastic condition, as the tracer remained mainly at the site of injection, with poor uptake in inguinal nodes and no evidence of dermal backflow (see also patients 5 and 6). Other findings included bicuspid aortic valve, diagnosed at the age of 28. A small conjunctival angioma was also observed in the right eye and a right frontal nevus, which appeared at 16 years of age, that was removed at 27.

Patient 5- L487P mutation

This woman, 34 years of age, developed lymphedema of the lower limbs at 26 years. Lymphedema was asymmetric, more severe on the left. Lymphoscintigraphy showed delayed passage of lymph on the left and early passage on the right. Inguinal lymph glands were visualised bilaterally, although on the left the tracer appeared only in the late phase. No dermal backflow was detected. The patient complained of more severe swelling on the left until December 2014; but subsequently she

developed severe swelling also of the whole right leg. In 2014, she underwent excision of a nevus on the right heel.

Patient 6- A492V mutation

This male patient, age 30 years, developed unilateral lymphedema of the left lower limb at 26 years. Lymphoscintigraphy showed slow lymphatic drainage of the left lower limb without anomalous lymph node stops. Poor scintigraphic visualisation of left inguinal and iliac lymph nodes, no visualisation of left lumbar lymph nodes, and no dermal backflow were detected. Slow lymphatic drainage of the right lower limb without anomalous lymph node stops or dermal backflow was observed. The patient presented normal scintigraphic visualisation of right inguinal and iliac lymph nodes.

Supplementary materials: Detailed methodological description of mutagenesis experiments reported in Mat and Met, paragraph "Cloning the *FOXC2* cDNA and generation of site-directed mutagenesis plasmids".

To introduce the c.826-827delAT mutation into NT-GFP-FOXC2, two site-directed mutagenesis reactions were made consecutively.

First mutagenesis:

The first PCR was performed using forward primer 5'-CAGCGTGGAGAACATCTGACCCTGCGAACGTC-3' and reverse primer 5'-GACGTTCGCAGGGTCAGATGTTCTCCACGCTG-3', which contain complementary sequence to the regions flanking the nucleotide 826, and competent cells were transformed with the amplification product. After TOP10 transformation, was selected a clone containing the NT-GFP-FOXC2(M276X) recombinant plasmid. After purification (PureLink Quick Plasmid Miniprep kit, Invitrogen), potential mutant construct was sequenced.

Second mutagenesis:

The NT-GFP-FOXC2(M276X) recombinant plasmid was used as template for the second site-directed mutagenesis reaction, in order to obtain the NT-GFP-FOXC2(M276DfsX186) vector. The primers designed for this amplification were: forward 5'- CAGCGTGGAGAACATCGACCCTGCGAACGTC-3' and reverse 5'-GACGTTCGCAGGGTCGATGTTCTCCACGCTG-3'. The potential clone was confirmed to contain the required mutation (c.826-827delAT) by a final sequencing

A

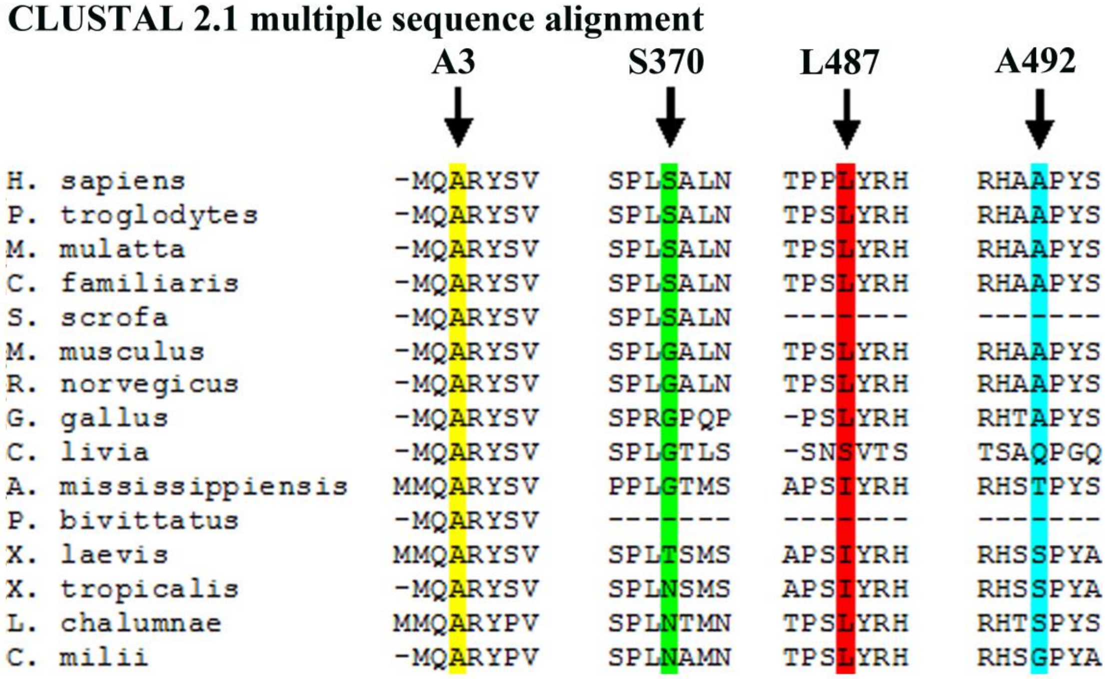

B

Missense Mutations	SIFT (score from 1 to 0. = 0.05 is damaging)	PolyPhen-2 (score from 0 to 1. = 0.8 is probably damaging)
**c.8C>G p.A3G**	**deleterious** (score 0)	**probably damaging** (score 0.999)
**c.1109G>C p.S370T**	**tolerated** (score 0.71)	**benign** (score 0.079)
**c.1460T>C p.L487P**	**deleterious** (score 0)	**probably damaging** (score 0.970)
**c.1475C>T p.A492V**	**deleterious** (score 0.01)	**benign** (score 0.164)

Supplementary Figure 1: Bio-informatic analysis of FOXC2 missense mutations. A) The amino acid sequence alignment of FOXC2 in 7 mammals (H. sapiens, P. troglodytes, M. mulatta, C. familiaris, S. scrofa, M. musculus and R. norvegicus,), 2 birds (G. gallus and C. livia), 2 reptiles (A. mississippiensis and P. bivittatus), 2 amphibians (X. laevis and X. tropicalis) and 2 fish (L. chalumnae and C.milii) reveals complete conservation of alanine 3 and partial conservation of serine 370, leucine 487 and alanine 492; multiple alignments were carried out with Basic GeneBee ClustalW version 2.1, web interface. FOXC2 protein NCBI accession number: NP_005242.1. B) Table reporting SIFT (http://sift.jcvi.org) and PolyPhen-2 (http://genetics.bwh.harvard.edu/pph2) prediction analysis of mutation score damage for p.A3G, p.S370T, p.L487P and p.A492V.

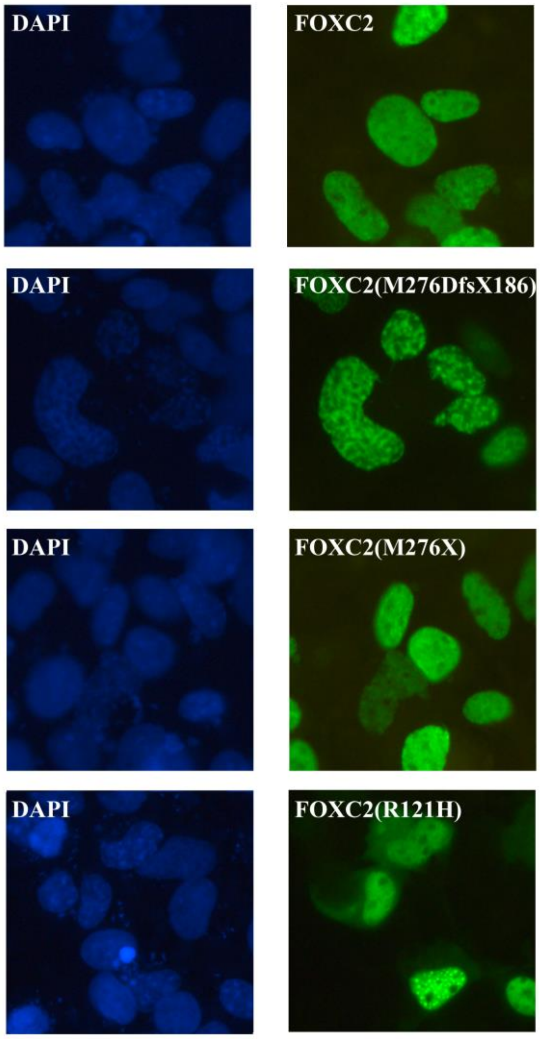

Supplementary Figure 2: Transient transfection of FOXC2 mutant proteins in COS7 cells. COS7 cells were transiently transfected with one of the following plasmids: pFOXC2-EGFP, pFOXC2(M276DfsX186)-EGFP, pFOXC2(M276X)-EGFP and pFOXC2(R121H)-EGFP. After 24 h, cells were fixed and stained with DAPI. The M276fs and R121H mutations caused FOXC2 protein aggregation, while pFOXC2-EGFP andpFOXC2(M276X)-EGFP displayed an homogeneous nuclear distribution. In green, FOXC2 proteins tagged with GFP and in blue, cellular nuclei stained with DAPI. Images were at 40X magnification.

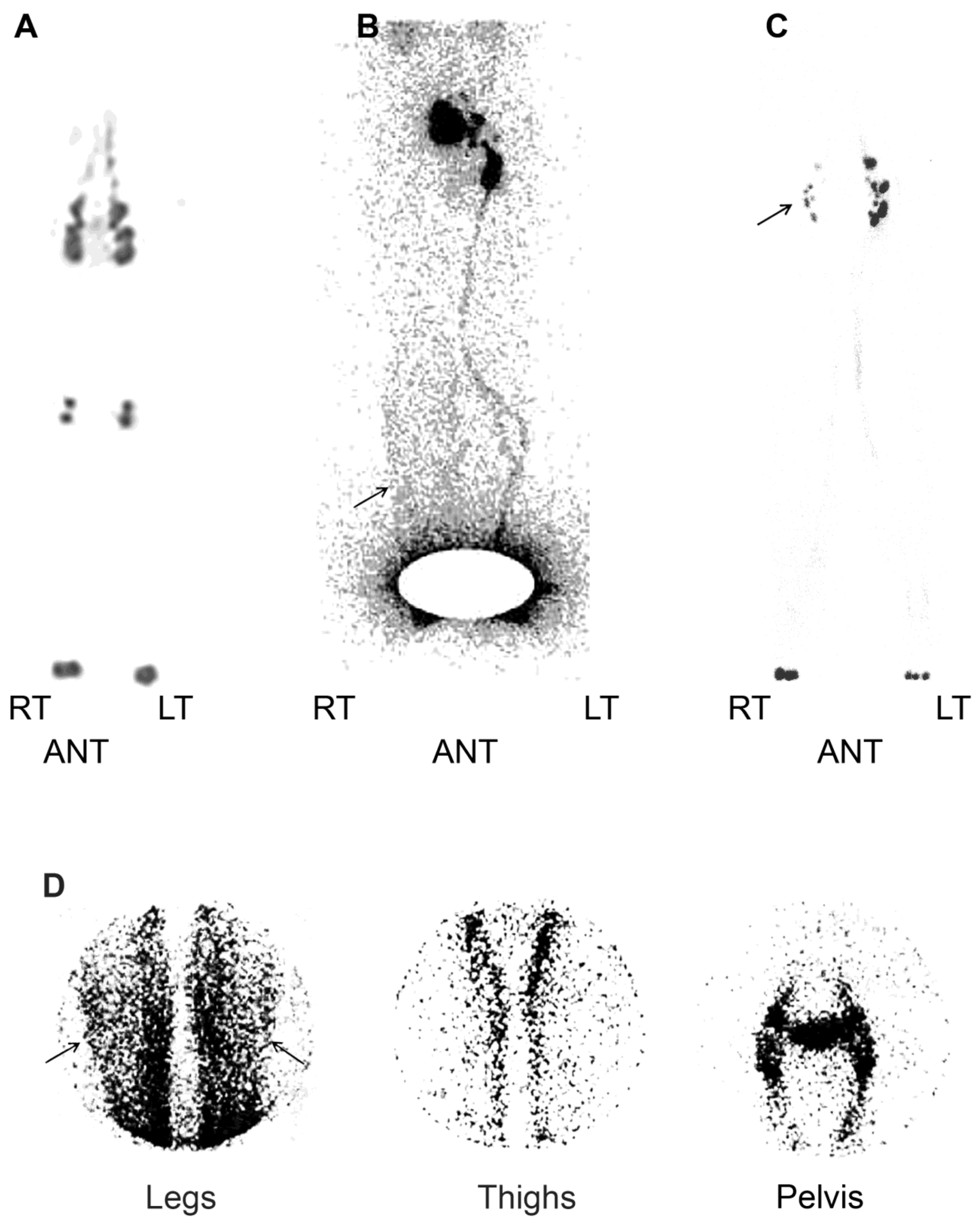

Supplementary Figure 3: Lymphoscintigraphy of a normal subject and LD patients. A) Normal uptake of radiocolloid tracer within lymphatic vessels was observed in a control subject; B) Lymphoscintigraphy image of patient 1 (A3G mutation: loss of function) showed a moderate slowing of radiocolloid return flow along lymphatic vessels of left lower limb and complete absence of visualisation of lymphatic ducts and lymph nodes of the right lower limb, associated with dermal backflow; C) Lymphoscintigraphy of patient 4 (Q420X mutation: gain of function) revealed bilateral slow transit of tracer, particularly on the right, and retention of tracer at the site of injection, without dermal backflow; indeed, scintigraphic imaging definition of the right inguinal, iliac and lumbar lymph nodes was reduced. D) Segmental lymphoscintigraphy of patient 2 (M276FfsX186 mutation: loss of function) showed lymph reflux in both legs, known as dermal backflow.

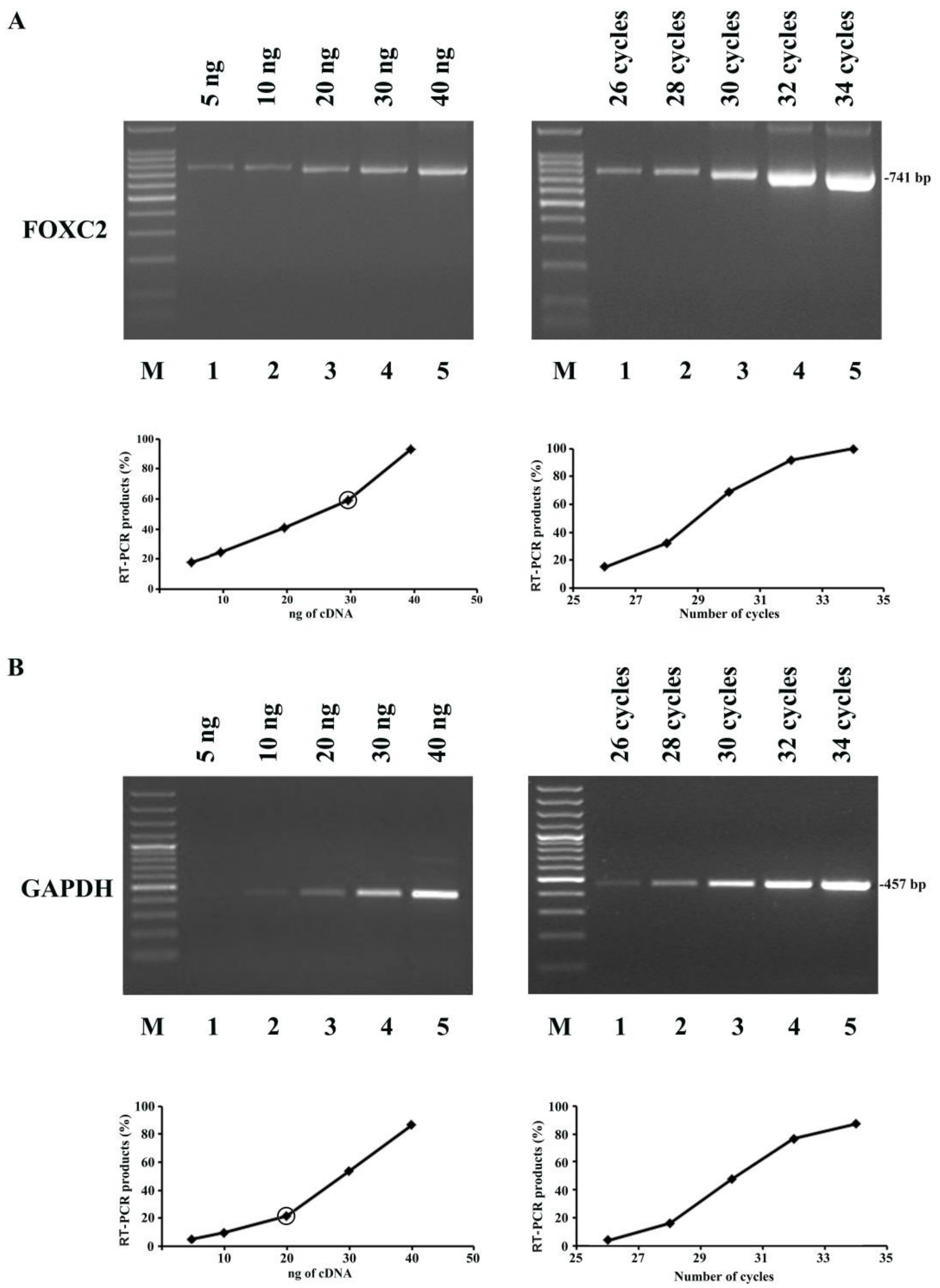

Supplementary Figure 4: Non saturating RT-PCR conditions for FOXC2 and GAPDH gene. RT-PCR gel analysis and representative regression curves showing the FOXC2 (A) and GAPDH (B) PCR products for different amounts of cDNAs and different number of cycles of amplification. Panel A on the left: evaluation of FOXC2 RT-PCR products from increasing amounts of cDNA (lanes 1-5); the empty black circle indicated the amount of cDNA (30 ng) utilized in the subsequent cycles kinetic experiment (lanes 1-5), showed on the right. Panel B on the left: evaluation of GAPDH RT-PCR products from increasing amounts of cDNA (lanes 1-5); the empty black circle indicated the amount of cDNA (20 ng) utilized in the subsequent cycles kinetic experiment (lanes 1-5), showed on the right. In all gels, Lane M refers to DNA molecular markers. For FOXC2 and GAPDH genes, the non-saturating conditions, were defined by data concerning the linear part of the curves. 30 ng of cDNA and 28 cycles have been utilized to perform the RT-PCR expression analysis of FOXC2 mutants reported previously in Figure 2A; while for GAPDH gene, 20 ng of cDNA and 28 cycles of amplification have been used (shown in Figure 2B).
